# Unilateral multifocal choroiditis following EBV-positive mononucleosis responsive to immunosuppression: a case report

**DOI:** 10.1186/s12886-020-01525-7

**Published:** 2020-07-09

**Authors:** P. P. Borkar, M. A. Grassi

**Affiliations:** 1 Department of Ophthalmology and Visual Sciences, University of Illinois at Chicago, 1905 W Taylor St, Chicago, IL 60612 USA; 2 Grassi Retina, Naperville, IL USA

**Keywords:** Multifocal choroiditis, Subretinal fibrosis, Immunosuppression, EBV, Mononucleosis, Schlaegel line, Unilateral, Azathioprine

## Abstract

**Background:**

Multifocal choroiditis (MFC) is a relatively uncommon bilateral inflammatory chorioretinopathy affecting Caucasian young women with myopia. We present images from a case of completely unilateral multifocal choroiditis following EBV-positive mononucleosis that demonstrated a dramatic clinical response to immunosuppression.

**Case presentation:**

A 20-year-old woman with bilateral high myopia (−6D) and a documented normal prior retinal examination presented with visual loss in the right eye 2 months following confirmed Epstein-Barr virus (EBV) positive mononucleosis. Ophthalmoscopic examination showed completely unilateral placoid lesions of variable age. The left eye was unaffected. Fluorescein angiography revealed active leakage, especially in the parafovea. Spectral domain optical coherence tomography (SD-OCT) demonstrated sub-retinal pigment epithelial nodular deposits, some of which were confluent with overlying intra-retinal fluid and indistinct margins. Upon treatment with the immunosuppressant azathioprine there was significant resolution of the lesions in her right eye along with improvement in vision.

**Conclusion:**

This is a rare case of completely unilateral MFC following an episode of EBV positive mononucleosis that showed a dramatic response to immunosuppression.

## Background

MFC is a relatively uncommon, chronic, bilateral inflammatory chorioretinopathy that predominantly affects young healthy myopic white women between the second and sixth decades of life with no known associated systemic disease or other recognized ocular syndromes [1]. It is characterized by inflammation at the level of the RPE and outer retina. MFC was first described in two young patients in 1973 by Nozik and Dorsch [2]. The term multifocal choroiditis and panuveitis was coined by Dreyer and Gass, who described 28 patients with uveitis and lesions at the level of the RPE and choriocapillaris [3]. It has been suggested that an association between MFC and Epstein–Barr virus (EBV) exists [4] but has never been proven.

## Case presentation

A 20-year-old woman presented with visual loss in the right eye. She had high myopia bilaterally (−6D). A normal prior retinal examination was documented 5 years prior to presentation. A routine contact lens exam 1 year prior to presentation recorded 20/20 vision. Review of systems was notable for confirmed EBV positive mononucleosis 2 months prior to the development of ocular symptoms. At that time, she had headaches, fatigue, sore throat and lymphadenopathy. No eye pain, redness or light sensitivity was noted. EBV mononucleosis was confirmed using the heterophile antibody (Monospot) test. The patient reported trouble with distance vision 2 months after the mononucleosis was diagnosed.

**Fig. 1:**
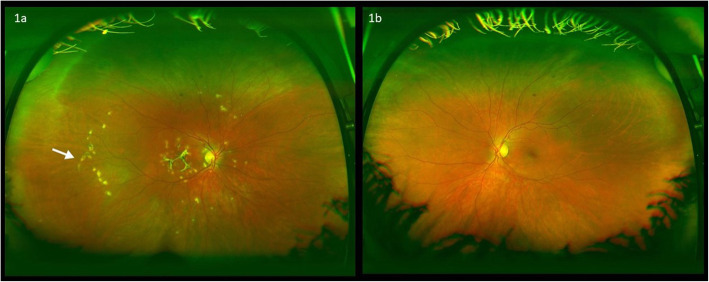
Ophthalmic examination revealed completely unilateral placoid lesions of variable age associated with an overlying fibrinous band in the macula and a peripheral Schlaegel line in right eye (Fig. 1a-arrow). The left eye was completely unaffected (Fig. 1b)

**Fig. 2:**
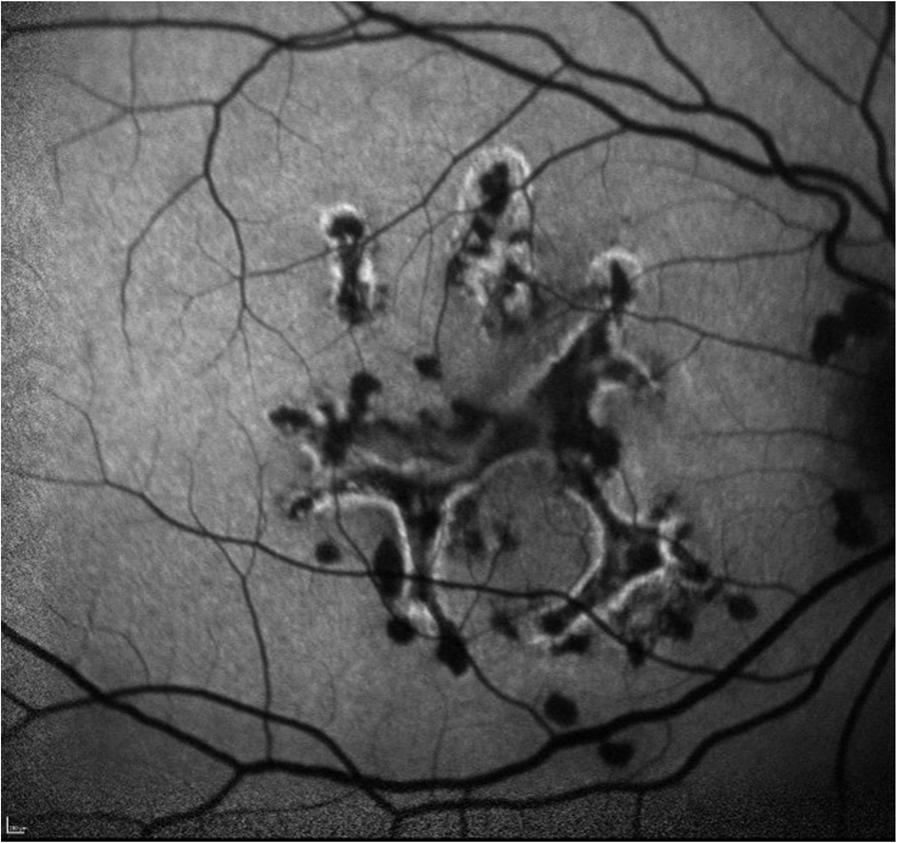
Fundus autofluorescence of the right eye revealing nummular hypofluorescent lesions, many of which have extensive areas of hyperfluorescence along their perimeters

**Fig. 3:**
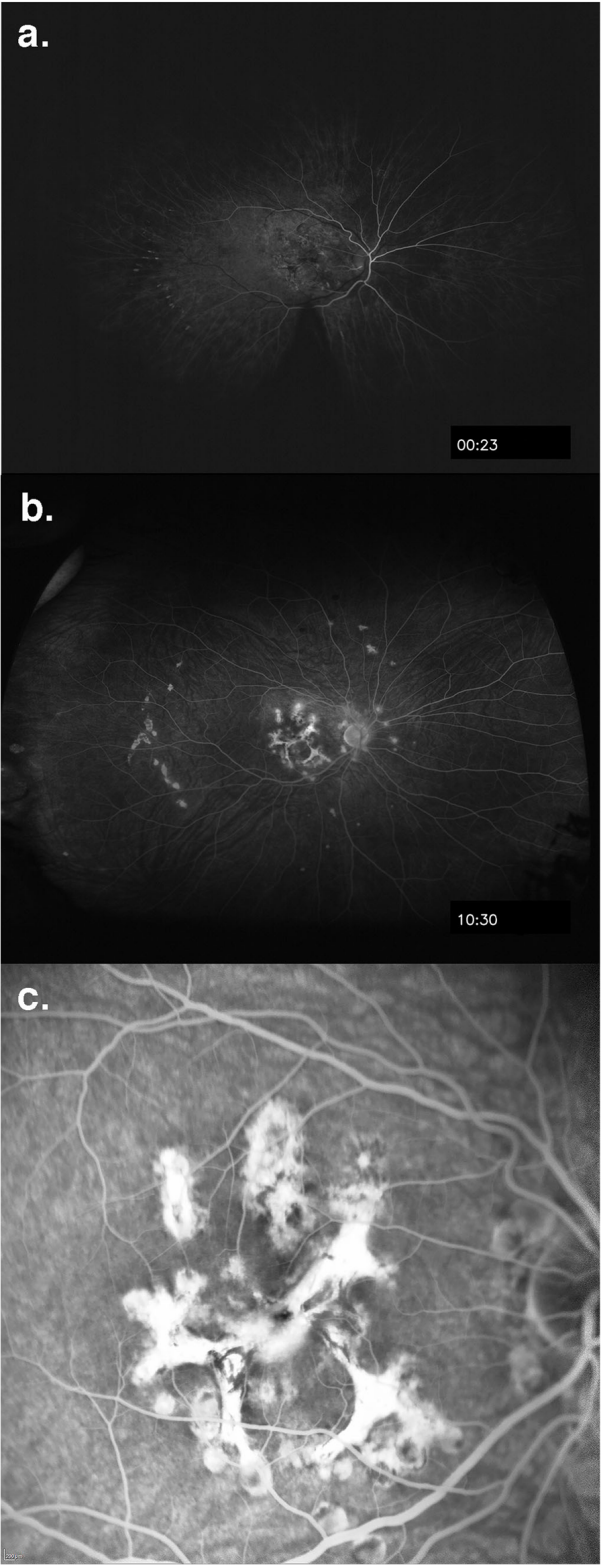
Early (**a**) and late (**b**) frames of fluorescein angiogram showing early hypofluorescence of the lesions. Blockage from both the lesions and pigment is evident. Transmission increase from the lesions in the mid-periphery is seen in the mid-phase. Several of the lesions that blocked early, demonstrate staining during the late phase of the angiogram. Magnified view (**c**)

**Fig. 4:**
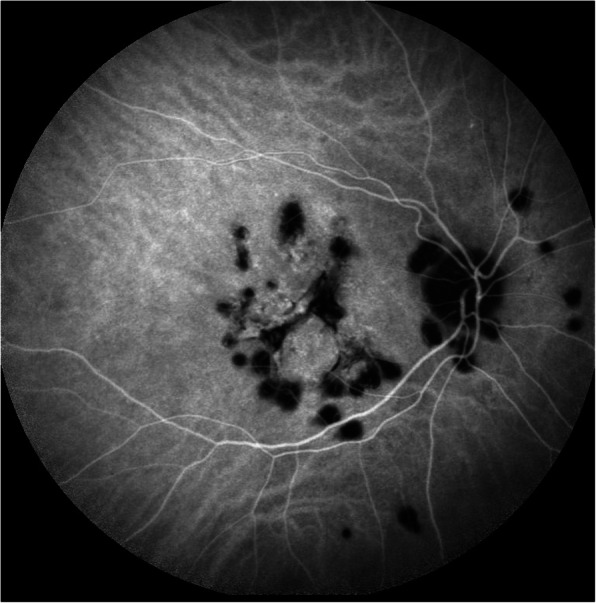
ICG angiogram showing hypofluorescent lesions in the right eye

**Fig. 5:**
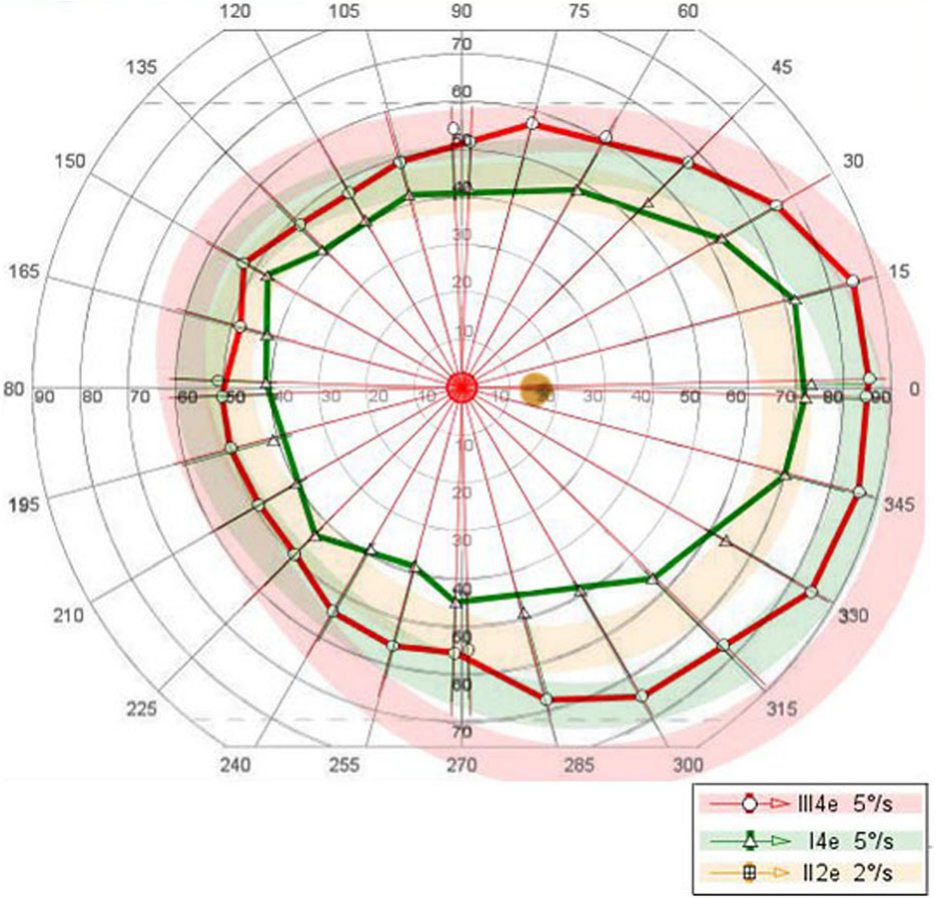
The overall global function of the right eye was excellent by kinetic perimetry

**Fig. 6:**
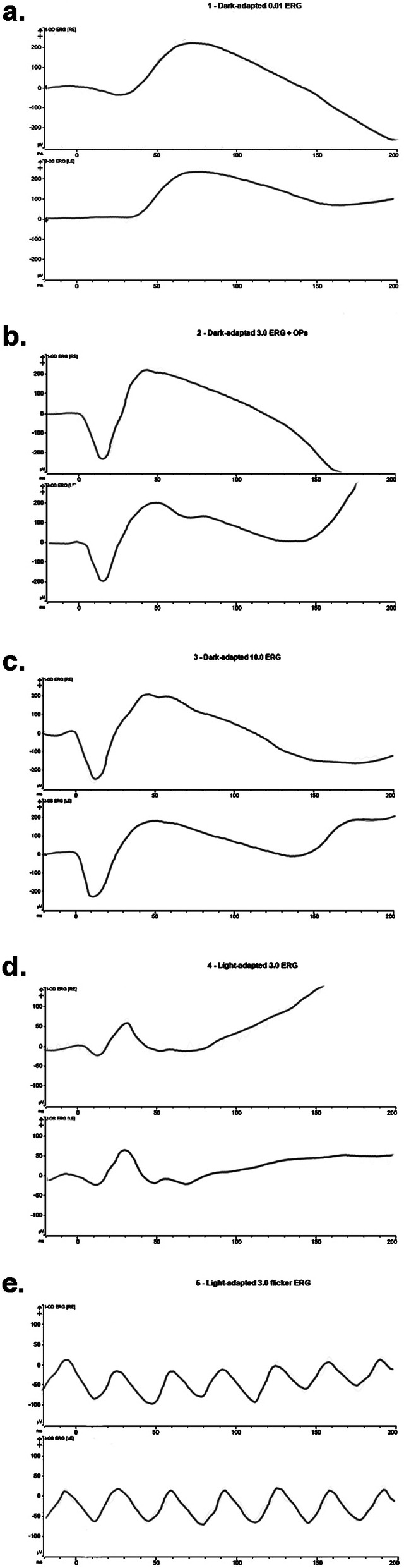
Full field electroretinogram (ERG) showing scotopic and photopic amplitudes and implicit times within normal limits

At presentation 6 months after her symptoms began ophthalmic examination of the right eye revealed quiet anterior chambers with scant anterior vitreous cells only in the right eye. Retinal examination showed punched out nummular chorioretinal lesions admixed with patchy areas of coarse pigmentation overlying subretinal fibrosis. The completely unilateral placoid lesions of variable age were associated with an overlying fibrinous band in the macula and a peripheral Schlaegel line (Fig. 1a-arrow). The left eye was pristine and completely unaffected (Fig. 1b). Fundus autofluorescence of the right eye revealed nummular hypofluorescent lesions, many of which had extensive areas of hyperfluorescence along their perimeters. (Fig. 2) Fluorescein angiography revealed active leakage, especially in the parafovea, suggestive of ongoing inflammation (Fig. 3a-c). ICG angiogram showed hypofluorescent lesions in the right eye on initial presentation (Fig. 4). SD-OCT demonstrated sub-RPE nodular deposits, some of which were confluent with overlying intraretinal fluid and indistinct margins. Overall global retinal function of the right eye was preserved as evident by kinetic perimetry (Fig. 5) and full field electroretinography (Fig. 6), suggesting a local rather than diffuse process.

Extensive systemic workup was performed. Given the history of EBV and the presence of sub-RPE deposits, lymphoma was ruled out with a chest CT scan. It only revealed reactive cervical lymph nodes. No hepatosplenomegaly was noted. Complete blood count with differential demonstrated a slight increase in lymphocytes (59.9% reference range 10–50%). The rest of the differential was within normal limits. RPR was non-reactive and the FT-ABS was negative. Interferon-Gamma release assay (QuantiFERON-TB gold) was negative. West Nile antibodies were negative, Brain MRI was negative for any CNS lesions or cerebral vasculitis. Liver enzymes were mildly raised: ALT 94 IU/L (reference range 7–52 IU/L) and AST 62 IU/L (reference range 15–40 IU/L). The pattern of elevated aminotransferases and lymphocytosis in the setting of EBV positivity was considered to be consistent with infectious mononucleosis [5].

**Fig. 7:**
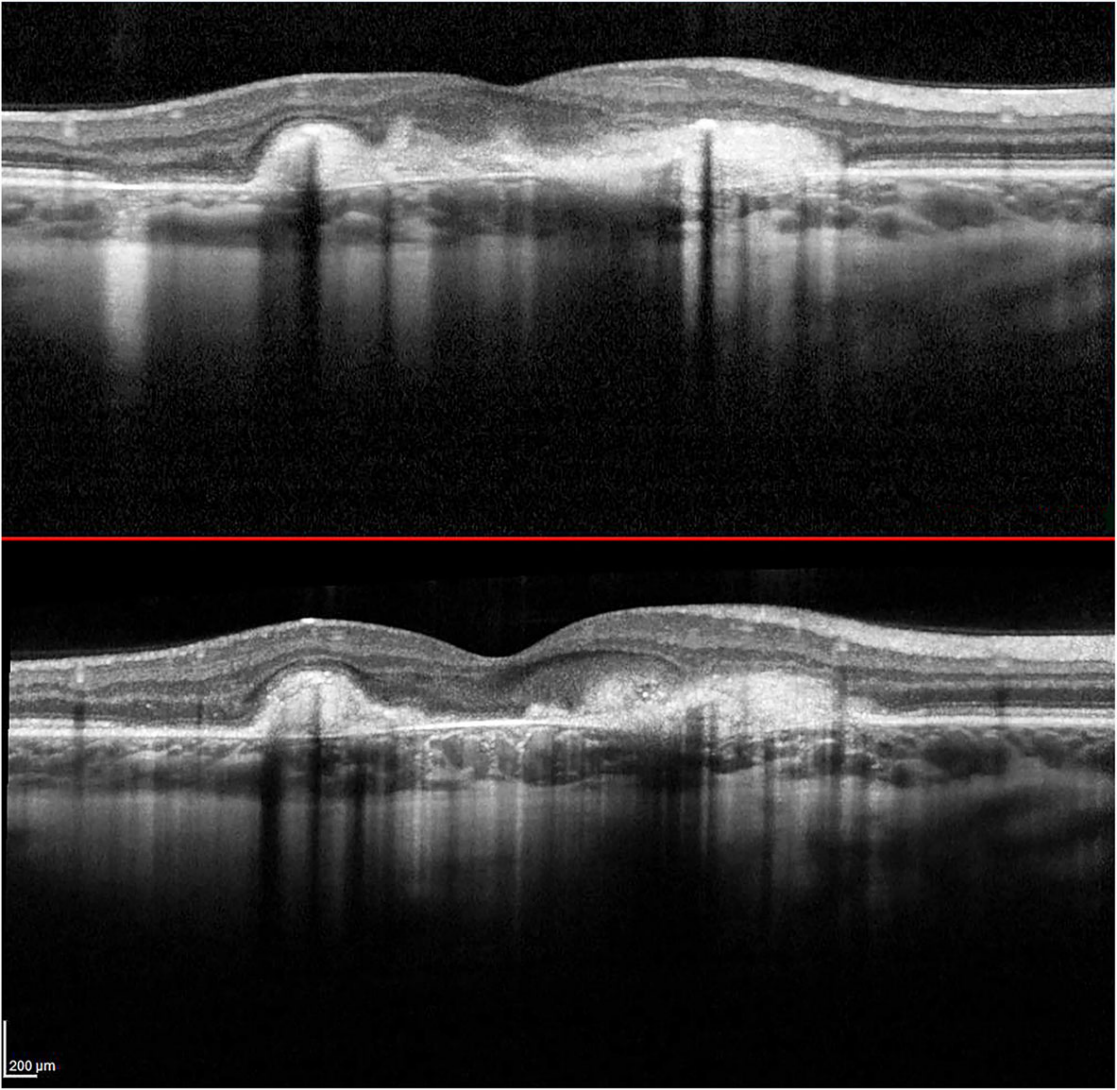
SD-OCT after treatment with azathioprine demonstrating consolidation of lesions and decreased inflammation

Given the activity of the MFC, the patient was started on an initial empiric high dose trial of prednisone. As the prednisone was tapered, she was switched to the immunosuppressant, azathioprine. There was significant interval resolution of the inflammatory nature of the lesions in the right eye. Over the following 8 months dramatic improvement was demonstrated resulting in consolidation of the subretinal lesions and decreased inflammation (Fig. 7). Vision correspondingly improved from 20/200 to 20/40. The left eye remains unaffected.

**Fig. 8:**
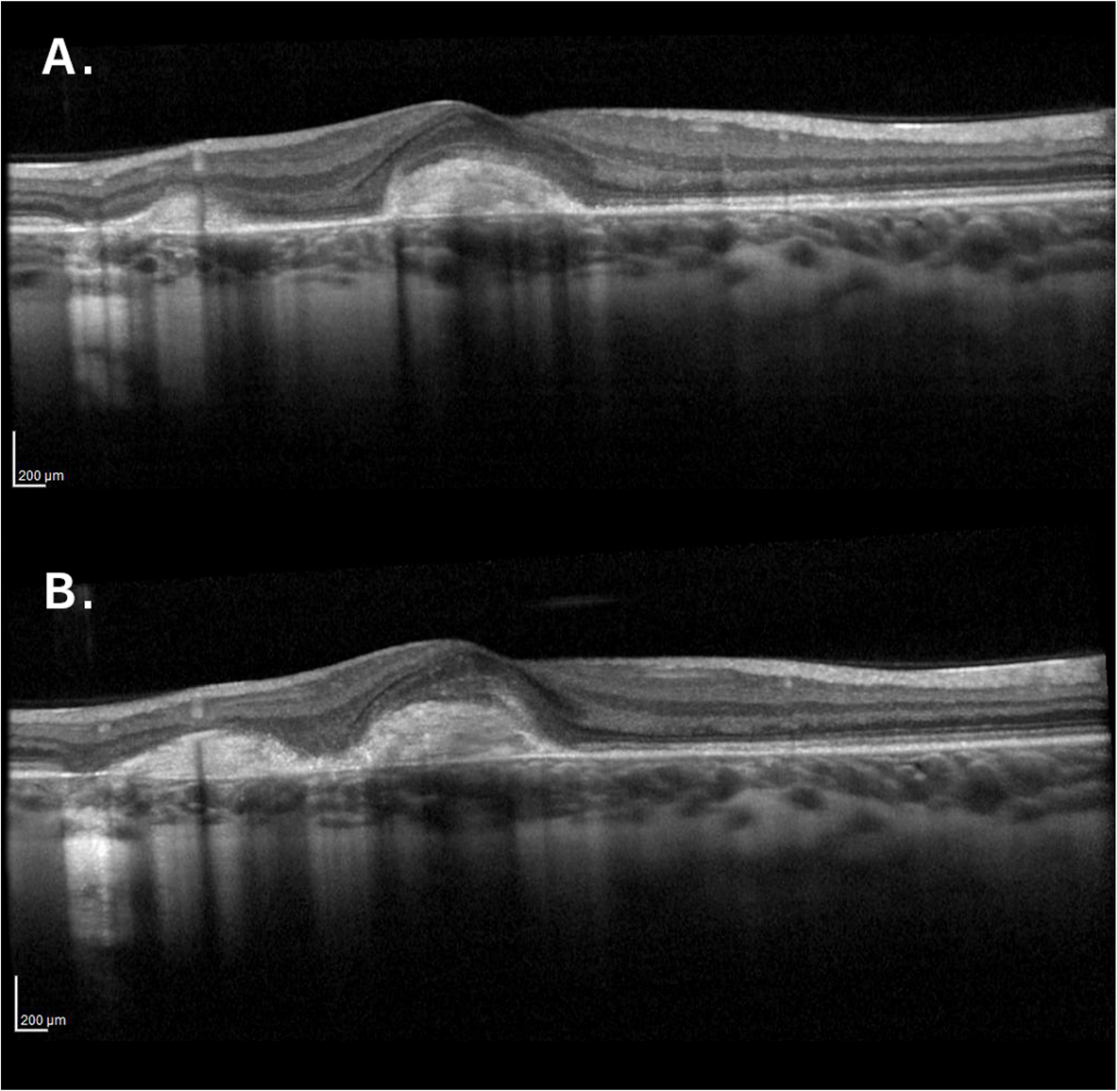
a and b. SD-OCT of the right eye 3 months apart showing increase in the activity of the multifocal lesions and new areas of inflammation after stopping the azathioprine

**Fig. 9:**
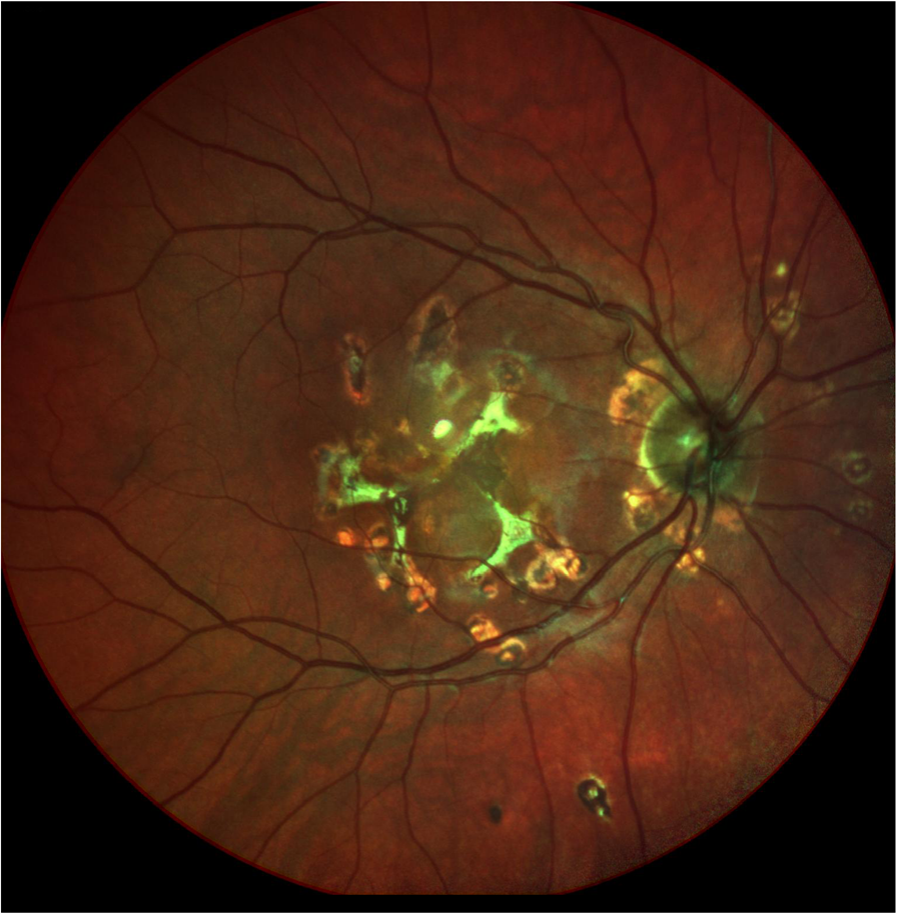
Fundus photo showing reduced chorioretinal inflammation and consolidation of lesions at 2 year follow up

During the patient’s course, she self-discontinued the azathioprine. During that three-month interval, there was an increase in the activity of the multifocal lesions evident on her exam and new areas of inflammation (Fig. 8) and a consequent drop in her vision. When azathioprine was resumed, these lesions resolved, and her vision improved. After 2 years of follow up, there has been continued evidence of reduced chorioretinal inflammation and consolidation of her retinal lesions on the azathioprine (Fig. 9). The left eye has fortunately remained pristine even after 2 years of follow up.

## Discussion and conclusions

Our patient’s prior history of EBV- positive mononucleosis was established with the heterophile antibody test which is an agglutination assay that detects anti-red blood cell antibodies produced as part of a polyclonal antibody response occurring during EBV infection. Heterophile antibody tests are rapid, cheap and specific tests with a result that is interpreted as positive or negative (for agglutination) and can be performed from the onset of symptoms of infectious mononucleosis [6, 7]. A positive i.e. reactive heterophile test in a patient with symptoms suggestive of IM is diagnostic of EBV infection [8] and further testing for specific antibodies to EBV is not considered essential.

The development of multifocal choroiditis may be one of the delayed sequelae of EBV infection. Chronic EBV infection has been conjectured to predispose to molecular mimicry and affect various major organ systems including the eye [9]. Interestingly, our patient had a documented prior normal retinal examination and 20/20 vision. She noted a change in her vision after the development of EBV associated IM. The complete unilaterality of her findings may explain the slight delay in her recognition of the visual changes. The development of MFC following confirmed EBV positive IM provides a temporal association between the EBV infection and her symptoms. The development of EBV-associated mononucleosis suggests a plausible sequence of events [10] in terms of EBV predisposing to or causing the multifocal choroiditis which has been conjectured previously [4].

Azathioprine is a steroid sparing immunosuppressant, mainly used in rheumatologic diseases. The patient had continuous improvement of her inflammatory lesions in the right eye after initiating azathioprine. The visual improvement was somewhat surprising, given the extent of associated fibrosis. The patient showed dramatic improvement in her retinal lesions over 8 months. It seems unlikely that this dramatic visual improvement over 8 months was unrelated to immunosuppression with azathioprine. She had 20/200 vision for a year with persistent active lesions before initiating azathioprine that completely subsided with vision improvement from 20/200 to 20/40 with immunosuppression. This course does not appear to follow the natural history of the disease [3], rather it suggests a response to intervention with immunosuppression. Long term visual prognosis in MFC may reasonably improve with continued immunosuppression, particularly in the event that persistent subclinical disease activity evolves into subretinal fibrosis and uveitis syndrome.

There is considerable value of titrating immunosuppression to the disease activity as evidenced by the reduced number of lesions along with improvement in vision with azathioprine immunosuppression. In fact, our patient demonstrated test-retest reliability when she self-discontinued the medicine (Figs. 8 and 9) This suggests that adequate immunosuppression may be causally responsible for her improvement and potentially the reason why her condition remained unilateral. Taken together, these findings argue that MFC progression and visual potential can be optimized through aggressive immunosuppression.

In summary, we present a completely unilateral case of MFC temporally correlated with EBV infection that demonstrated a dramatic improvement in vision and lack of involvement of the fellow eye with aggressive immunosuppression.

## Data Availability

Not applicable.

## Abbreviations

EBV: Epstein-Barr virus
MFC: Multifocal choroiditis
SD-OCT: Spectral domain optical coherence tomography
RPE: Retinal pigment epithelium
CT: Computed tomography
ICG: Indocyanine green angiography
FA: Fluorescein angiography
IM: Infectious mononucleosis

## References

[1] Morgan CM, Schatz H (1986). Recurrent multifocal choroiditis. Ophthalmology.

[2] Nozik RA, Dorsch W (1973). A new chorioretinopathy associated with anterior uveitis. Am J Ophthalmol.

[3] Dreyer RF, Gass DJ (1984). Multifocal choroiditis and panuveitis. A syndrome that mimics ocular histoplasmosis. Arch Ophthalmol.

[4] Tiedeman JS (1987). Epstein-Barr viral antibodies in multifocal choroiditis and panuveitis. Am J Ophthalmol.

[5] Brigden ML (1999). Infectious mononucleosis in an outpatient population: diagnostic utility of 2 automated hematology analyzers and the sensitivity and specificity of Hoagland's criteria in heterophile-positive patients. Arch Pathol Lab Med.

[6] Marshall-Andon T, Heinz P (2017). How to use … the Monospot and other heterophile antibody tests. Arch Dis Child Educ Pract Ed.

[7] Basson V, Sharp AA (1969). Monospot: a differential slide test for infectious mononucleosis. J Clin Pathol.

[8] Evans AS (1975). A prospective evaluation of heterophile and Epstein-Barr virus-specific IgM antibody tests in clinical and subclinical infectious mononucleosis: Specificity and sensitivity of the tests and persistence of antibody. J Infect Dis.

[9] Straus SE (1988). The chronic mononucleosis syndrome. J Infect Dis.

[10] Lucas RM, McMichael AJ (2005). Association or causation: evaluating links between “environment and disease”. Bull World Health Organ.

